# Genome-wide association study combined with biological context can reveal more disease-related SNPs altering microRNA target seed sites

**DOI:** 10.1186/1471-2164-15-669

**Published:** 2014-08-08

**Authors:** Di Wu, Gang Yang, Lifang Zhang, Jiwei Xue, Zhining Wen, Menglong Li

**Affiliations:** College of Chemistry, Sichuan University, Chengdu, 610064 P.R. China

**Keywords:** microRNA, Genome-wide association study, Single nucleotide polymorphisms, Human diseases and cancers

## Abstract

**Background:**

Emerging studies demonstrate that single nucleotide polymorphisms (SNPs) resided in the microRNA recognition element seed sites (MRESSs) in 3′UTR of mRNAs are putative biomarkers for human diseases and cancers. However, exhaustively experimental validation for the causality of MRESS SNPs is impractical. Therefore bioinformatics have been introduced to predict causal MRESS SNPs. Genome-wide association study (GWAS) provides a way to detect susceptibility of millions of SNPs simultaneously by taking linkage disequilibrium (LD) into account, but the multiple-testing corrections implemented to suppress false positive rate always sacrificed the sensitivity. In our study, we proposed a method to identify candidate causal MRESS SNPs from 12 GWAS datasets without performing multiple-testing corrections. Alternatively, we used biological context to ensure credibility of the selected SNPs.

**Results:**

In 11 out of the 12 GWAS datasets, MRESS SNPs were over-represented in SNPs with p-value ≤ 0.05 (odds ratio (OR) ranged from 1.1 to 2.4). Moreover, host genes of susceptible MRESS SNPs in each of the 11 GWAS dataset shared biological context with reported causal genes. There were 286 MRESS SNPs identified by our method, while only 13 SNPs were identified by multiple-testing corrections with a given threshold of 1 × 10^−5^, which is a common cutoff used in GWAS. 27 out of the 286 candidate SNPs have been reported to be deleterious while only 2 out of 13 multiple-testing corrected SNPs were documented in PubMed. MicroRNA-mRNA interactions affected by the 286 candidate SNPs were likely to present negatively correlated expression. These SNPs introduced greater alternation of binding free energy than other MRESS SNPs, especially when grouping by haplotypes (4210 vs. 4105 cal/mol by mean, 9781 vs. 8521 cal/mol by mean, respectively).

**Conclusions:**

MRESS SNPs are promising disease biomarkers in multiple GWAS datasets. The method of integrating GWAS p-value and biological context is stable and effective for selecting candidate causal MRESS SNPs, it reduces the loss of sensitivity compared to multiple-testing corrections. The 286 candidate causal MRESS SNPs provide researchers a credible source to initialize their design of experimental validations in the future.

**Electronic supplementary material:**

The online version of this article (doi:10.1186/1471-2164-15-669) contains supplementary material, which is available to authorized users.

## Background

MicroRNAs are small non-coding RNAs ~22 nt which are involved in key biological processes such as cell proliferation, cell apoptosis and metabolism [1, 2]. Aberrant expression level of microRNAs has been demonstrated to be associated with common diseases and human cancers [3, 4]. MicroRNAs achieve their biological function by binding to mature mRNAs and decreasing the expression level of target mRNAs in most cases. Mechanisms behind microRNA target recognition have been well-studied, and the most stable feature is the nucleotides 2–8 in 5′-end of microRNAs, which is so-called ‘seed’ region. Normally, seed region of a microRNA exactly matches to the complementary sequence in 3′UTR of its target mRNA, which is usually called ‘microRNA recognition element seed site’ (MRESS) following Watson-Crick base pairing. SNPs in both microRNA seed region and 3′UTR MRESS can interfere with target recognition, either by eliminating/impairing/consolidating an existing microRNA-mRNA interaction or by creating a new interaction. However, in view of the observations that one microRNA can regulate hundreds of mRNAs and one mRNA may have multiple microRNA target sites in its 3′UTR [5–7], SNPs residing in the 3′UTR MRESSs should be the more common genetic factor affecting the binding between microRNAs and mRNAs. Allele-specific mRNA expression induced by MRESS SNPs is proved to be associated with several diseases and cancers: Nicoloso *et al*. [8] observed genotype AG carriers of rs334348 G > A suffered higher risk of breast cancer (AG vs. AA, OR = 1.69, p = 0.048), whereas rs334348 located in a MRESS of miR-628-5p in 3′UTR of the TGFBR1 gene and G-allele consolidates target-binding. Expression level of TGFBR1 protein degraded after transfecting of miR-628-5p and differed by genotype of rs334348 (50% and 20% for GG and AA respectively, p = 0.006). Wang *et al*. [9] reported rs12720208 C > T in a MRESS of miR-433 in the 3′UTR of FGF20 gene as a causal SNP of Parkinson’s disease (risk allele: T, p = 0.0019). Risk allele T can introduce a G:U wobble in the binding site and impair target binding. They transfected luciferase reporter gene combined with 3′UTR of FGF20 into Neuro2A cells that expressed miR-433, and observed a ‘dramatically’ reduction of luciferase signal for reporter gene with C-allele while reporter gene with T-allele did not. Pathological analysis reveals positive correlation between expression level of FGF20 and α-Synuclein, whereas overexpression of α-Synuclein is previously reported to cause PD, which implies pathogenesis of the T-allele is to abolish the interaction between FGF20 gene and miR-433 thus lead to up-regulation of α-Synuclein.

About 60% of protein-coding genes are putative microRNA targets [10] and millions of MRESSs may reside in 3′UTR of these genes, which makes exhaustively experimental validation for the causality of MRESS SNPs impractical. Some researchers have tried using genome-wide association study (GWAS) to solve this problem, taking advantage of its ability to detect susceptibility of hundreds of thousands of SNPs simultaneously. Richardson *et al*. [11] identified 87 MRESS SNPs in strong linkage disequilibrium (LD) (r^2^ > 0.8) with a collection of highly susceptible SNPs (p ≤ 1 × 10^−5^) in NHGRI GWAS catalog [12]. Significance of the observation was 1.08 × 10^−23^. Further analysis provided microRNA-mRNA co-expression evidence for 39 of these MRESS SNPs and 11 were also supported by eQTL mapping. Thomas *et al*. [13] proposed a method to score the effect of MRESS SNPs basing on a two-step SVM classifier and took haplotype into account. They linked scored MRESS SNPs to 2112 top-ranking SNPs from a GWAS of breast cancer by LD and observed positive correlation between effect score and LD degree. Liu *et al*. [14] developed an online database to annotate predicted MRESS SNPs. They searched a list of 7705 susceptible SNPs obtained by a GWAS of schizophrenia against the MRESS SNPs collected by their database. Three meet stringent significance threshold of p ≤ 1.0 × 10^−5^, which indicated true positive association existed in MRESS.

Despite the achievements have obtained in previous studies, limitations still remain to be improved. Existing methods only focus on the subset of MRESS SNPs with p-value less than a stringent multiple-testing corrections threshold (usually p ≤ 1 × 10^−5^) in GWAS datasets in order to control false positive results. However, many true positive results will be eliminated in exchange. In addition, biological context of MRESS SNPs such as the pathogenesis of their host genes and corresponding microRNAs have been neglected. A MRESS SNP with just an extremely significant p-value or a high effect score does not necessarily work by interfering with microRNA targeting. Similarly, a MRESS SNP that disrupts target recognition may not be very striking in statistics. Moreover, previous studies covered only a few GWAS datasets (no more than 3 datasets) at a time. The feasibility of selecting causal MRESS SNPs by GWAS analysis (say p-value or odds ratio) still needs to be tested under more GWAS datasets. Here, we proposed a method which integrated GWAS p-value, gene ontology (GO) annotation and pathogenesis of human disease to select causal MRESS SNPs without performing multiple-testing corrections. The method was applied to 12 GWAS datasets covering 11 common diseases and cancers of human. Results demonstrated MRESS SNPs are active disease regulators in most of the GWAS datasets. Literature-based causality query, co-expression evidence and alternation of binding free energy also proved that our method is plausible. Finally, a list of 286 candidate causal MRESS SNPs with adequate credibility was provided for further experimental validation. About 90% of them were newly reported.

## Results and discussion

### MRESSs in 3′UTR of coding-mRNAs are under purifying selection

**Figure 1:**
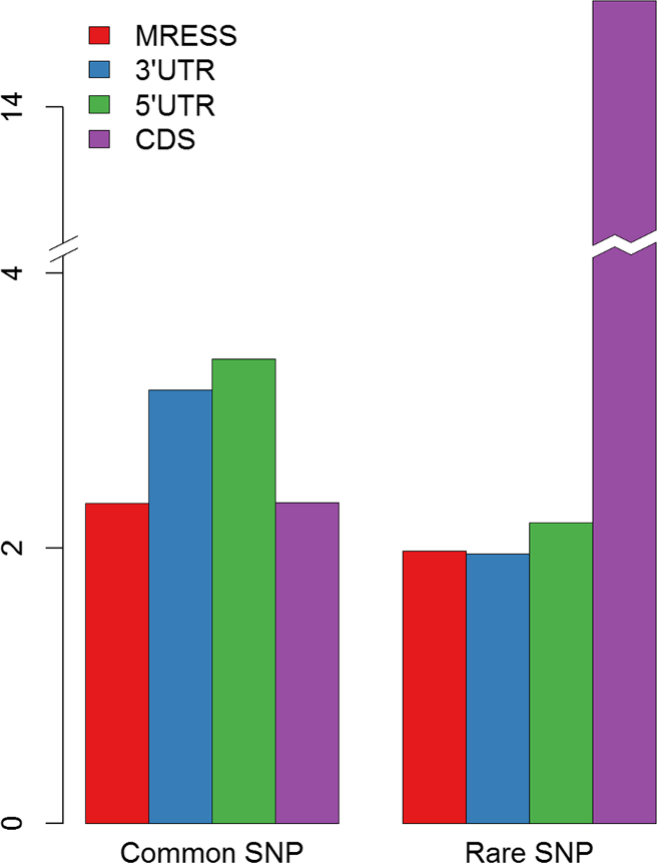
**Comparisons of the SNP density between different mRNA regions grouped by global MAF.** SNPs with global MAF ≥ 0.01 are defined as common SNPs, the rest are rare SNPs. The figure illustrates that the MRESS region has a significantly lower common SNP density but almost the same rare SNP density compared to the UTR regions, which indicates the MRESS region is under strong purifying selection. (Of note, the exceptionally high rare SNP density of the CDS region is derived from its unparalleled sequencing coverage).

Evolutionary conserved regions are thought to be functional, and the conservation of microRNA targets has been reported [10]. Previous studies always compared the SNP density of MRESSs with their flanking region, but horizontal comparison between MRESS and other mRNA region, such as CDS, has not been implemented. Moreover, they took all SNPs into account at a time. However, common SNPs (minor allele frequency (MAF) ≥ 0.01) and rare SNPs (MAF < 0.01) affected human diseases in different patterns [15, 16]. It’s more rational to calculate the density of these two kinds of SNPs separately. We collected experimentally validated microRNA-mRNA interactions from miRecord [17] and mirTarbase [18], and predicted their MRESSs in 3′UTR by TargetScan6.1 [19]. Obtaining MRESSs were thought to be adequately credible. Then, we mapped genomic coordinates of all the bi-allelic SNPs in dbSNP135 to that of the predicted MRESSs and thus calculated SNP density. We detected 312 MRESS SNPs in total, comprising of 212 SNPs lacking population frequency, 54 common SNPs and 46 rare SNPs (Of note, SNPs with <100 observed chromosomes were considered as lacking frequency data since they cannot accurately generate MAF value to differentiate common and rare SNPs). Distinct results emerged for common SNPs and rare SNPs after comparison of SNP density in different mRNA regions (Figure 1). On the one hand, the density of common SNPs in MRESSs was significantly less than that of the 3′UTRs and the 5′UTRs, even as low as that of the CDSs, which indicated MRESSs were highly conserved. On the other hand, the density of rare SNPs in MRESS was very close to that of the 3′UTRs and the 5′UTR. The contrast of densities between common and rare MRESS SNPs demonstrated MRESSs were under strong purifying selection and MRESS SNPs were deleterious.

### MRESS SNPs interfere with microRNA-regulated human diseases

**Figure 2:**
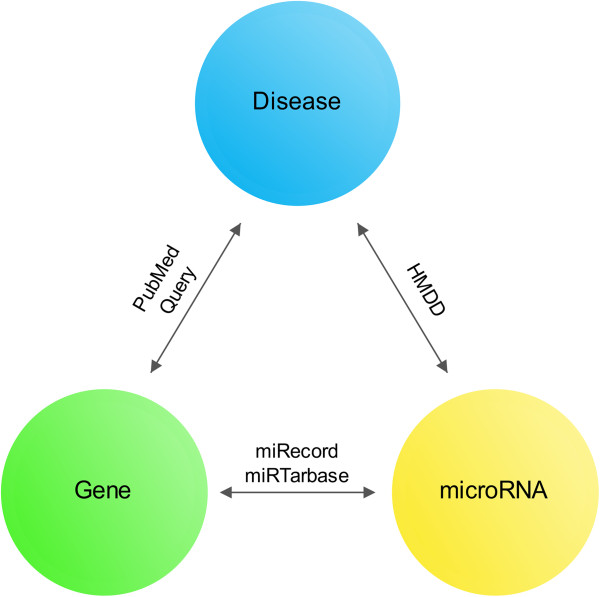
**Schematic diagram for the construction of the microRNA-gene-disease three-way interactions.** Ways to build up relationships between any 2 of the 3 elements are marked above the double-headed arrows. If a microRNA and a gene share the same related disease, we believe that their interaction is also associated with the disease, and any factor such as MRESS SNP that can interfere with this interaction is highly susceptible.

Association between microRNA and human disease was established if aberrant microRNA expression presented in patient group. Since most MRESS SNPs were embedded in 3′UTR of mature mRNAs but not genomic regions associated with microRNA biogenesis, they should not be the principal factors behind abnormal microRNA expressions. However, microRNAs regulated human diseases by interacting with mRNAs ultimately. Therefore association between microRNA and human disease can be converted to association between microRNA-mRNA interaction and human disease, giving MRESS SNPs the potential to affect microRNA-regulated human diseases. Based on this assumption, we generated trilateral interactions for microRNA, gene and disease, including microRNA-gene interactions obtained from miRecord and mirTarbase, microRNA-disease interactions obtained from HMDD database [20] and gene-disease interactions obtained from PubMed (Figure 2). For all the established trilateral interactions, we believed that the microRNA-gene interactions are disease-related since both part of the interaction were associated with the same disease. Finally, we constructed association between 2109 validated microRNA-gene interactions and 352 human diseases. 281 MRESS SNPs have the potential to affect these interactions and thus lead to disease. We searched the 281 MRESS SNPs in PubMed and 11 had been reported to be deleterious. Reported diseases associated with these 11 MRESS SNPs were highly concordant with those associated with their microRNA-mRNA interactions (Table 1), which supported our assumption that MRESS SNPs can interfere with microRNA-regulated human diseases.

**Table 1:** **Comparison of the reported diseases between 11 validated MRESS SNPs and their corresponding interactions**

SNP ID	Interaction	Reported disease of SNP	Reported disease of interaction
rs1042538	IQGAP1::hsa-miR-124	Breast cancer*	Breast neoplasms*/prostatic neoplasms/colorectal neoplasms…
rs1054190	NR1I2::hsa-miR-148a	Primary sclerosing cholangitis**	Hepatocellular carcinoma**/leukemia…
rs1056628	MMP9::hsa-miR-491-5p	Atherosclerotic cerebral infarction*	Cerebral infarction*/multiple sclerosis…
rs1057233	SPI1::hsa-miR-569	Systemic lupus erythematosus*	Systemic lupus erythematosus*
rs1063320	HLA-G:: hsa-miR-152	Multiple sclerosis**/systemic lupus erythematosus/pre-eclampsia*/HCV infection	Atherosclerosis**/pre-eclampsia*/asthma…
rs11574744	HNF4A:: hsa-miR-34a/34c-5p	Diabetes**/renal cell carcinoma*	Metabolic diseases**/renal cell carcinoma*/coronary artery disease/alcoholic fatty liver…
rs12720208	FGF20:: hsa-miR-433	Parkinson’s disease*	Parkinson’s disease*/ovarian neoplasms…
rs1621	MET:: hsa-miR-199a-3p	Chronic rhinosinusitis	Atrophic muscular disorders/Crohn’s disease…
rs16917496	SETD8:: hsa-miR-502-5p	Breast cancer/small-cell lung cancer/*epithelial ovarian cancer/*hepatocellular carcinoma	Ovarian neoplasms*/hepatocellular carcinoma*
rs28521337	NTRK3:: hsa-miR-485-3p	Anxiety disorders	Asthma/hepatocellular carcinoma/leukemia…
rs5186	AGTR1:: hsa-miR-155	Renal disease*/hypertension*/inflammation*/pre-eclampsia*/type 2 diabetes*/mountain sickness/diastolic heart failure/*hypertrophic cardiomyopathy**/aldosterone-producing adenoma**	Renal insufficiency**/kidney diseases*/hypertension*/inflammation*/pre-eclampsia*/diabetes mellitus*/diabetes complications/heart failure*/adenocarcinoma**…

*Diseases that exactly matched between the two blanks.

**Diseases that matched at a broad level (e.g. diabetes vs. metabolic diseases).

### GWAS analysis reveals enrichment of MRESS SNPs in susceptible variants

Experimentally validated microRNA-mRNA interactions account for only a small part of the real relationships. So we performed a genome-wide target prediction to identify more possible MRESS SNPs in 3′UTR of protein-coding mRNAs. We predicted MRESS for both wild type and mutant type 3′UTR sequences and divided all of them into 7mer (1–7), 7mer (2–8), 8mer-1a and 8mer sites by the patterns of seed match. Only MRESSs conserved between human and mice were retained to limit false positive interactions. Finally, 150,301 3′UTR SNPs embedded in predicted MRESSs were classified according to their contributions to the interactions (Table 2).

**Table 2:** **Classification of the 150,301 predicted MRESS SNPs**

Contribution	Number of MRESS SNPs			
	7mer (1–7)	7mer (2–8)	8mer-1a	8mer
Create	64,672	51,127	18,147	33,111
Loss	64,490	51,716	17,712	33,275

**Figure 3:**
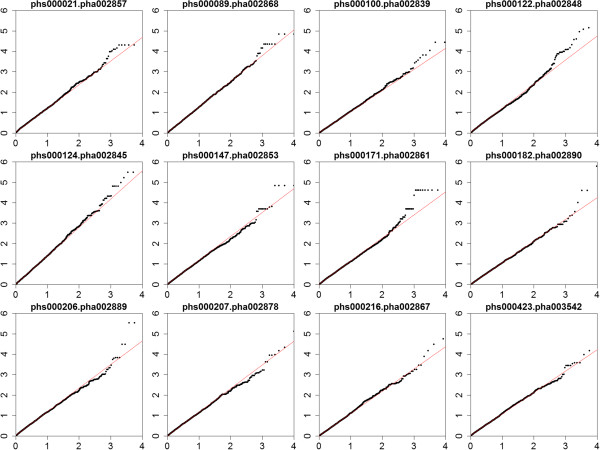
**Quantile-Quantile plot for MRESS SNPs in all the 12 GWAS datasets.** The units for both x-axis and y-axis are –log10(p). We can observe upward deviation from the red line in most of the subplot, which is the clue for true positive signals, and imply MRESS SNPs as common factors involved in multiple diseases and cancers.

To evaluate the effects of these predicted MRESS SNPs on human diseases, we downloaded the analytical results of 12 GWAS datasets of European-ancestry populations covering 11 human diseases and cancers. We utilized the ‘proxy search’ tool provided by SNAP database [21] to select GWAS-SNPs having high LD with the predicted MRESS SNPs in CEU population, then assigned the p-values of GWAS-SNPs to their corresponding MRESS SNPs as measurement of susceptibility. Finally, a total number of 12,892 predicted MRESS SNPs were mapped to at least one GWAS-SNP in any of the 12 datasets and presented in subsequent analysis. According to the hypothesis of ‘common disease, common variant’, common diseases are polygenic diseases manipulated by multiple common variants embedded in coding or regulatory regions. Therefore the effect size of a single causative variant should be modest [22]. In addition, microRNA regulations are spatio-temporal specific, which means only a part of the microRNA-mRNA interactions are active in the diseased tissue. Taking these two points into consideration, we assumed that p-values of the MRESS SNPs are enriched in the susceptible part of each of the GWAS datasets if true association existed, but majority of the functional MRESS SNPs would not meet stringent multiple-correction threshold because of their modest effective size. To validate our assumption, we drew Quantile-Quantile plot for each of the 12 GWAS datasets (Figure 3). In most of the 12 datasets, obviously upward deviation from theoretical distribution at the right side was observed, and only 13 MRESS SNPs had –log10(p) larger than 5, which is a general threshold of significance in GWAS, demonstrating the reasonability of our assumption.

### GWAS p-value combined with biological context identifies hundreds of candidate causal MRESS SNPs

**Figure 4:**
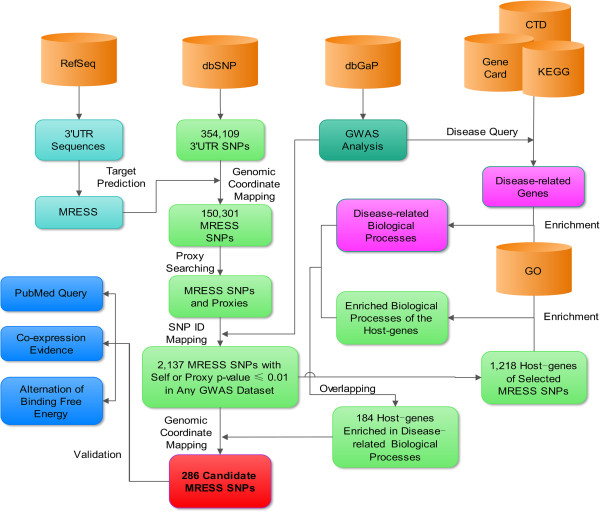
**Workflow of our method for selecting candidate causal MRESS SNPs from multiple GWAS datasets.** We integrated functional annotations from GO database to filter out false positive MRESS SNPs rather than to perform multiple-correction. The obtained causal SNPs are greatly increased. We further validated our results by three different approaches. The results proved our method stable and effective.

We have discovered a hint for true association signals between MRESS SNPs and human diseases from the analysis above (Figure 3), and the next step is to distinguish them from background signals and filter out false positive. Multiple-testing correction is not a good option because it impairs sensitivity greatly. MRESS SNPs have the potential to regulate microRNA-induced mRNA repression. If they are embedded in functional genes which act on interested disease, the positive signals should be more credible, and a moderate p-value threshold should meet the needs. According to this guideline (Figure 4), we first set up a moderate significance threshold of p ≤ 0.01 and selected out 2,116 MRESS SNPs. Next, we conducted GO-term enrichment for the 1,218 host genes of these MRESS SNPs in each of the GWAS datasets and got 12 lists of enriched biological processes. Meanwhile, we collected disease-related genes for each of the GWAS datasets from CTD [23], KEGG [24] and Gene Card [25] and also got 12 lists of enriched biological processes. At last, we did one-by-one comparison between the two sets of lists, found their degree of overlap remarkably higher than expected. For example, in dataset phs000122.pha002848 which is related to systemic lupus erythematous (SLE), 102 genes contain at least one significant MRESS SNPs and are enriched in 70 biological processes, *e.g.* positive regulation of tissue remodeling (p = 2.8 × 10^−5^), inflammatory response (p = 0.0050), B-cell activation (p = 0.018) and T-cell activation (p = 0.040). In these biological processes, 21 overlap with the enriched biological processes of genes that have been reported to be associated with SLE. Significance of this observation is 3.3 × 10^−20^, demonstrating the existence of the true association signals. We got similar results from 10 other GWAS datasets except for a GWAS dataset studied age-related macular degeneration (Table 3).

**Table 3:** **Statistics of the 12 GWAS datasets**

Dataset	Disease	#Enriched GO-terms	#Disease-related GO-terms	Fisher-p	#Candidate SNPs	#PubMed SNPs	Fisher-p
phs000021.pha002857	Schizophrenia	48	9	0.0003	49	3	0.05
phs000089.pha002868	Parkinson’s disease	49	7	0.001	22	2	0.05
phs000100.pha002839	Type 2 diabetes	29	8	8 × 10^−5^	27	3	0.009
phs000122.pha002848	Systemic lupus erythematosus	70	21	4 × 10^−20^	19	7	4 × 10^−7^
phs000124.pha002845	Neuroblastoma	92	5	0.003	28	4	0.001
phs000147.pha002853	Breast cancer	26	12	6 × 10^−6^	29	2	0.08
phs000171.pha002861	Multiple sclerosis	56	3	0.02	17	2	0.03
phs000182.pha002890	Age-related macular degeneration	42	0	1	0	0	1
phs000206.pha002889	Pancreatic cancer	27	13	3 × 10^−9^	51	3	0.05
phs000207.pha002878	Prostate cancer	46	26	7 × 10^−14^	36	2	0.1
phs000216.pha002867	Systemic lupus erythematosus	23	3	0.009	7	3	2 × 10^−4^
phs000423.pha003542	Coronary artery disease	19	4	2 × 10^−4^	17	0	

‘#Enriched GO-terms’ denotes the number of enriched GO-terms for the host genes of MRESS SNPs with p-value ≤ 0.01 in each dataset. ‘#Disease-related GO-terms’ denotes the number of enriched GO-terms shared between the host genes and the disease-related genes collected from CTD, KEGG and Gene Card databases in each dataset. The probability of obtaining such number of disease-related GO-terms is calculated by fisher exact test. ‘#Candidate SNPs’ denotes the number of MRESS SNPs passing our selection standards in each dataset, and ‘PubMed SNPs’ denotes how many of them are reported to be deleterious by PubMed query, the probability of the observation is also calculated by fisher exact test. We can figure out from the table that most of the p-values are below the significant level of 0.05, which indicates MRESS SNPs as causal variants in most of the GWAS datasets.

In total, we found 184 host-genes of significant MRESS SNPs enriched in overlapped biological processes with disease-related genes. The 286 MRESS SNPs (see Additional file 1: Table S1) embedded in these genes with p-value ≤ 0.01 were selected as candidate causal MRESS SNPs.

### Candidate MRESS SNPs are over-represented in known deleterious 3′UTR SNPs

To access the effectiveness of our method for selecting causal MRESS SNPs, we retrieved PubMed for all the 12,892 MRESS SNPs with proxy in GWAS datasets and defined 368 of them with at least one query result as positive samples (In view of the foreseeable importance of the MRESS region, we removed SNPs that have been reported to be in MRESS to suppress the priori bias). Positive samples distributed un-evenly in different seed types. MRESS SNPs embedded in 7mer (2–8) sites possess the greatest precision (19/165 = 0.12), followed by 7mer (1–7) sites (16/201 = 0.080), 8mer sites (8/109 = 0.073) and 8mer-1a sites (3/62 = 0.048) respectively. 8mer and 8mer-1a sites possess larger binding energy than 7mer sites and are tolerable to mismatch. It may explain their relatively low precision. In total, 27 candidate MRESS SNPs (Table 4) are positive, the sensitivity and precision of our method are 0.073 (27/368) and 0.094 (27/286) respectively. In contrast, only 2 in all the 13 MRESS SNPs with p-value ≤ 1 × 10^−5^ are positive. The sensitivity of multiple-testing correction is much lower than our method (0.0054 vs. 0.073) whereas its precision doesn’t present significant advantage (0.15 vs. 0.094). The probability of getting 27 unbiased, PubMed-documented SNPs in 286 randomly selected MRESS SNPs is 5.0 × 10^−8^, which indicates candidate causal MRESS SNPs are in truly functional regions. We also calculated this probability for each of the GWAS datasets and 8 of them showed statistical significance (p ≤ 0.05, Table 3). Taking the SLE dataset phs000122.pha002848 as an example again, 7 of 19 candidate causal MRESS SNPs are known deleterious SNPs passing correction, significance of the observation is 4 × 10^−7^. The seven SNPs are rs2741918, rs450021, rs1057972, rs10954213, rs13317, rs1049623 and rs1042032, respectively. To speculate their pathogenesis, we performed detailed literature analysis for each of the seven SNPs.

**Table 4:** **Information about the 23 candidate causal MRESS SNPs have been reported to be deleterious**

SNP ID	Allele	MAF	Type	Gene	microRNA	Disease	p-value	#Reference
rs1053005	A > G	0.30	Create	STAT3	hsa-miR-4793-5p	Multiple sclerosis	0.007	2
rs1053023	A > G	0.29	Loss	STAT3	hsa-miR-4506/4640-5p	Multiple sclerosis	0.007	4
rs2116830	C > A	0.09	Create/Loss	KCNMA1	hsa-miR-2052/659	Multiple sclerosis	0.005	1
rs2057482	T > C	0.23	Create	HIF1A	hsa-miR-196a/196b/3174/3927/921	Neuroblastoma	0.002	4
rs5177	C > G	0.32	Loss	LRP8	hsa-miR-526b	Neuroblastoma	0.003	2
rs2272383	A > G	0.44	Create	TUB	hsa-miR-450a	Breast cancer	0.001	3
rs1349265	T > C	0.50	Create	THRB	hsa-miR-1468	Pancreatic cancer	0.003	1
rs788173	G > A	0.40	Create	DLX1	hsa-miR-4330	Pancreatic cancer	0.002	2
rs3751934	G > T	0.47	Create	RPTOR	hsa-miR-1231/4667-5p/4700-5p/637	Parkinson’s disease	0.002	1
rs7309	G > A	0.43	Loss/Create	TANK	hsa-miR-3941/466/4789-3p	Parkinson’s disease	0.0005	1
rs61865882	T > C	0.08	Create	EGR2	hsa-miR-499-3p/499a-3p	Prostate cancer	0.01	1
rs3160	T > C	0.38	Loss	MLST8	hsa-miR-329/362-3p/603	Prostate cancer	0.01	1
rs1064395	G > A	0.26	Create	NCAN	hsa-miR-1205/3665/4418/509-3-5p/509-5p/657	Schizophrenia/neuroblastoma	0.005	3
rs2296135	A > C	0.44	Create/Loss	IL15RA	hsa-miR-2276/4766-5p	Schizophrenia	0.002	2
rs5177	G > C	0.32	Loss	LRP8	hsa-miR-526b	Schizophrenia	2 × 10^−5^	2
rs1042032	A > G	0.43	Loss	EPHX2	hsa-miR-183	Systemic lupus erythematosus	0.003	2
rs1049623	A > G	0.46	Create	DDR1	hsa-miR-4499/4513	Systemic lupus erythematosus	8 × 10^−5^	1
rs1057972	A > T	0.48	Create/Loss	IL15	hsa-miR-4514/4692/940/1207-5p	Systemic lupus erythematosus	0.004	2
rs10954213	G > A	0.47	Create/Loss	IRF5	hsa-miR-543/4477a/4729/181a/664	Systemic lupus erythematosus	0.0007	21
rs11466285	T > C	0.08	Create	TGFA	hsa-miR-3190	Systemic lupus erythematosus	0.009	2
rs1217412	G > A	0.32	Create/Loss	PTPN22	hsa-miR-380/4495/3668	Systemic lupus erythematosus	0.005	1
rs13317	T > C	0.28	Loss	FGFR1	hsa-miR-3128/4470	Systemic lupus erythematosus	0.0008	3
rs2741918	C > T	0.38	Create	MEFV	hsa-miR-1226/4252/4733-3p	Systemic lupus erythematosus	0.005	1
rs450021	C > A	0.37	Create	MEFV	hsa-miR-4435/4645-5p/4673/4755-3p	Systemic lupus erythematosus	0.005	1
rs12613	G > A	0.08	Create	CBS	hsa-miR-3664-5p/3944-5p	Type 2 diabetes	0.0002	2
rs2336219	G > A	0.25	Create	ERCC1	hsa-miR-4698/545/548p	Type 2 diabetes	0.001	1
rs735482	A > C	0.34	Create	ERCC1	hsa-miR-4475	Type 2 diabetes	0.001	5

‘A > G’ denote allele A as the reference allele and G as the variant allele. The ‘Type’ column denotes the effect of the variant allele to the given microRNA-gene interactions, a type of ‘Create/Lose’ means the variant allele of the SNP can create some of the given interactions while eliminate others.

Rs2741918 C > T and rs450021 C > A are SNPs with high LD, and both are located in the MRESSs of MEFV gene. Mutant alleles T and A create the interactions between MEFV mRNA and miR-1226/4252/4733-3p as well as MEFV mRNA miR-4435/4645-5p/4673. Ustek *et al*. [26] reported higher frequency of heterozygotes for the two SNPs in the familial Mediterranean fever (FMF) patients after removing interference of CDS SNPs (75% vs. 48.5%, p = 0.006, OR = 3.2), while Notarnicola *et al*. [27] demonstrated MEFV gene was down-regulated in FMF patients. The observations are concordant with the speculation that mutant alleles of the two SNPs create microRNA binding sites and lead to repression of the MEFV gene. Furthermore, Shinar *et al*. [28] found mutations in MEFV gene affect phenotypes of SLE patients, such as SLE onset (earlier), renal failure (decrease) and febrile episodes (more common). Rs1057972 A > T is located in MRESS of IL15 gene. Mutant allele T interrupts binding-site of miR-1207-5p while creating new binding-sites for miR-940/4514/4692. Pistilli *et al*. [29] discovered that genotype AT and TT of rs1057972 are associated with pre- to post-training absolute difference in 1RM strength (elevate, p = 0.02), fasting glucose (decrease, p = 0.03) and BMI (decrease, p = 0.008), which means mutant allele T leads to the development of muscle strength. Combining with the report that IL15 can inhibit protein degradation [30] and counter muscle loss, which is a clinical feature of SLE patient [31]. Allele T may achieve its function through abolishing the repression of IL15 mRNA by miR-1207-5p, thus resulting in up-regulation of IL15 protein. Rs10954213 G > A is located in MRESS of IRF5 gene. Mutant allele A impairs interactions between IRF5 mRNA and miR-181a/664 while enhancing binding-site of IRF5 and miR-543/4477a/4729. Liu *et al*. [32] and Hu *et al*. [33] validated the association between rs10954213 and SLE in multi-ethnic population using meta-analysis. Studies of Alonso-Perez *et al*. [34] and Lofgren *et al*. [35] demonstrated mutant allele A induces up-regulation of IRF5 mRNA and IRF5 protein in SLE patients. Another observation reported by Lashine *et al*. [36] is the significant down-regulation of miR-181a in SLE patients. All these evidences strongly imply that rs10954213 regulates SLE by altering the binding of IRF5 mRNA and miR-181a. Rs13317 T > C is located in MRESS of FGFR1 gene. Mutant allele C eliminates interactions between FGFR1 and miR-3123/4470. Guimaraes *et al*. [37] reported the association between rs13317 and fracture non-union while study of Jun *et al*. [38] showed higher frequency of allele C in ossification of the posterior longitudinal ligament patients (p = 0.048, OR = 1.35). It was also reported that up-regulation of FGFR1 gene leads to joint fusion in mouse [39]. Taking all the three reports into account, the microRNA dysfunction induced by mutant allele C should be the mechanism behind injury of bone and ligament, which is also a manifestation of SLE patient. Rs1049623 A > G is located in MRESS of DDR1 gene. Mutant allele G creates interactions between DDR1 mRNA and miR-4499/4513. Roig *et al*. [40] revealed the association between rs1049623 and schizophrenia. Mutant allele G corresponds to the down-regulation of DDR1 gene in peripheral blood lymphocytes of schizophrenia patients, which is consistent with the role of G allele that introduces microRNA repression. Note that some SLE patients have been reported to suffer from schizophreniform psychosis [41], and polymorphisms at the locus of rs1049623 may be a plausible reason. Rs1042032 A > G is located in EPHX2 gene. Mutant allele G causes loss of the interaction between EPHX2 mRNA and miR-183. Lee *et al*. [42] observed higher risk of renal dysfunction after kidney-transplantation in individuals carrying genotype AA of rs1042032 (p = 0.04). Hama *et al*. [43] reported an obvious down-regulation of EPHX2 gene in mouse liver isografts compared with allografts (FC = 3.29), which implies down-regulation of EPHX2 is associated with transplant rejection. As genotype AA maximizes the repression of EPHX2 induced by miR-183. Its pathogenic mechanism may have a rational explanation.

In summary, experimental evidences of all the seven candidate causal MRESS SNPs support the inference that they are functional SNPs with the abilities to mediate microRNA-induced mRNA repression, and it’s worth noting that some of them have not been reported to be associated with SLE directly, but are related to other diseases that share the same or similar symptoms with SLE, indicating MRESS SNPs as common pathogenic factor beyond specific disease.

### Candidate MRESS SNPs tend to be embedded in negatively co-expressed microRNA-mRNA interactions and change binding free energy of the MRESSs greatly

**Figure 5:**
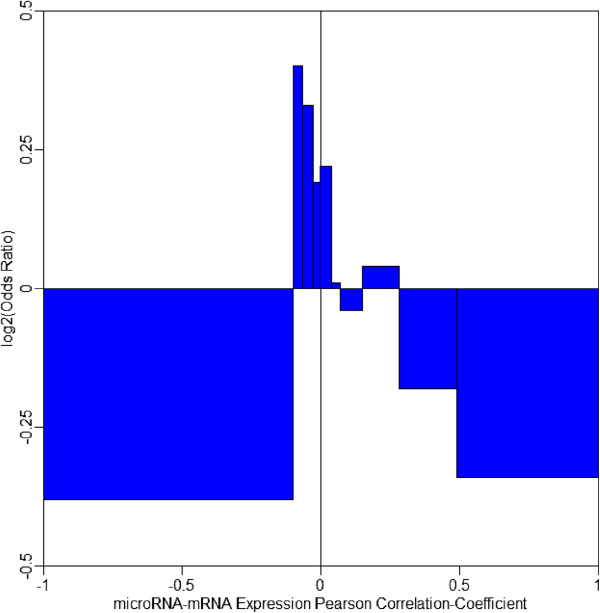
**Enrichment pattern of Pearson correlation coefficients of the co-expressed microRNA-mRNA pairs containing candidate causal MRESS SNPs.** We divided pairs with candidate causal MRESS SNPs into ten aliquots according to the correlation coefficients and obtained ten intervals. Then, we counted the number of MRESS SNPs, not necessarily candidate SNPs, falling into each interval. At last, we divided the number of candidate causal MRESS SNPs in each interval by that of the total set and took logarithm. A value greater than zero means microRNA-mRNA pairs containing candidate causal MRESS SNPs enrich in this part, and a value less than zero stands for anti-enrichment. This figure displays significant enrichment at parts of weakly negative correlation and anti-enrichment at parts of positive correlation, which demonstrates pairs containing candidate causal MRESS SNPs are more likely to be interactive.

Co-expression of microRNA and mRNA in the same tissue is the prerequisite of their interaction. If further evidence demonstrates their co-expression is negatively correlated, regulatory relationship can be established. We retrieved mirCoX database and obtained co-expression evidences for 495 microRNA-mRNA interactions affected by the candidate causal MRESS SNPs. Then, we compared their Pearson correlation coefficient to that of the other interactions predicted by TargetScan. Significant enrichment at the part of weakly negative correlation and anti-enrichment at the part of positive correlation were observed for these interactions (Figure 5). Correlation coefficients were not enriched in the part of highly negative correlation probably because most of the 495 interactions were created/enhanced by minor allele of the candidate causal MRESS SNPs. For the majority of the samples included by mirCoX database, such interactions may only maintain at a relatively low level. Another reason would be the mix of expression from all tissues that dilutes the truly negative correlations in real diseased tissues.

**Figure 6:**
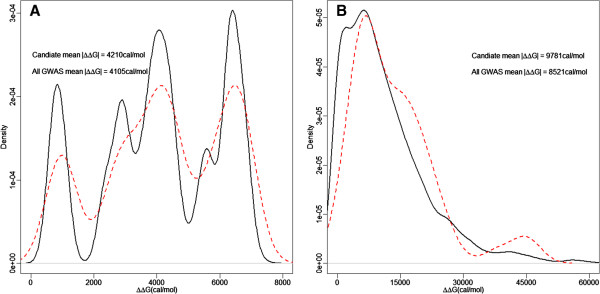
**Distribution of the alternations of binding free energy (ΔΔG) are different between candidate causal MRESS SNPs and the total set of MRESS SNPs in 12 GWAS datasets.** Red dotted line denotes the candidate causal MRESS SNPs while black solid line denotes the total set of MRESS SNPs in 12 GWAS datasets. ΔΔG in Figure 6 **A** is calculated by single SNP while that in Figure 6 **B** is calculated by haplotype. We can observe a decline of the peak near zero and rightward shift for candidate MRESS SNPs, which indicates the greater alternation of binding free energy are created.

MRESS SNPs will change the affinity of MRESSs and thus lead to alternation of binding free energy between wild type and mutant type MRESSs. Candidate SNPs should trigger greater energy alternation to ensure effectiveness. To test this hypothesis, we calculated alternation of binding free energy (ΔΔG) for each MRESS SNP using a nearest-neighbor algorithm [44]. Then we compared ΔΔG of candidate MRESS SNPs to that of the total set (Figure 6A). A slightly decline of the peak near zero was observed for candidate causal MRESS SNPs, which was concordant with a larger average |ΔΔG| of 4210 cal/mol for candidate causal MRESS SNPs versus 4105 cal/mol for all MRESS SNPs mapped to GWAS datasets. This result proved our hypothesis.

A single mRNA may have multiple MRESSs in its 3′UTR. If the SNPs embedded in these MRESSs are in high LD with each other, their effects can be accumulated as haplotype scores. Haplotypes with greater accumulated |ΔΔG| should have greater impact on the expression level of mRNAs. We identified 3970 haplotypes comprised of at least two MRESS SNPs in 3′UTR of 1336 protein-coding genes, using LD data of 1000 genome pilot1. The average |ΔΔG| is 8521 cal/mol. If we only concern about haplotypes containing candidate MRESS SNPs, the average |ΔΔG| would increase to a much greater value of 9781 cal/mol. We also compared the distribution of ΔΔG between haplotypes containing candidate MRESS SNPs and the total set, and got more striking results (Figure 6B).

## Conclusion

MicroRNAs are important post-transcriptional regulators. Variants in their recognition element seed sites can result in dysfunction of microRNA-induced mRNA repression and thus lead to diseases. In this study, we proved MRESS SNPs to be functional for both credible MRESSs and predicted MRESSs. For credible MRESSs, we first demonstrated they were under strong purifying selection, and then proved that MRESS SNP acted through interfering with microRNA-mRNA interaction. For predicted MRESSs, we did in-depth analysis for p-value of MRESS SNPs in 12 GWAS datasets and found indication of truly disease-associated signals in most of datasets. We also proposed a method to select candidate causal MRESS SNPs based on both their p-values in GWAS datasets and the relevance between their host genes and the interested disease. Our method effectively reduced the loss of true positive signals caused by multiple-testing corrections, and had the potential to speculate pathogenesis of interested MRESS SNP because of the introduction of biological context. Candidate causal MRESS SNPs obtained by our method were over-represented in the known deleterious 3′UTR SNPs. They tended to be embedded in negatively co-expressed microRNA-mRNA interactions and generated greater binding free energy alternation. All of these evidences proved our method, and the perception of selecting causal variant using GWAS information is stable and effective. At last, the 286 candidate causal MRESS SNPs provided future researchers with a good source to initialize their studies.

## Methods

### Retrieval and processing of microRNA & mRNA data

We downloaded microRNA data from miRBase version 17 [45], and then abstracted all the 1539 human microRNAs with prefix ‘hsa-’ but not ended up with an asterisk. We set up two sets of seed sequence for microRNAs, using base 1–7 and 2–8 respectively from 5′-end of their mature sequences. For each set, microRNAs with the same seed were grouped together in order to save computing time. Sequences of mRNAs and their annotation data were retrieved from NCBI RefSeq and NCBI Genome databases respectively. The ‘human.rna.fna.gz’ file from RefSeq database contains well-curated and unique sequences of human mature mRNAs, while the ‘seq_gene.md’ file from Genome database provides genome coordinates and region annotations of the mRNAs collected in the ‘human.rna.fna.gz’ file. We integrated data from these two files using a Perl script and thus obtained sequences and genome coordinates of 29,962 3′UTR, 24,785 5′UTR and 31,375 CDS. As protein-coding genes are primary targets for microRNAs, we only retained mRNAs with the prefix ‘NM_’. The sequences and genome coordinates are both GRch37.p10 version.

### Retrieval and processing of SNP information

We retrieved SNP data from the file ‘snp135.txt.gz’ provided by the UCSC genome browser [46], which deposited annotation data of all the SNPs collected by dbSNP version 135, including genome coordinate, alleles and allele frequencies. For the sake of convenience, we only retained bi-allelic SNPs located in autosomes and sex chromosome X. Mapping between SNPs and mRNAs/MRESSs was conducted by R package ‘BSgenome’ using genome coordinates. A total of 1,149,607 SNPs are mapped to the mature mRNAs regions, of which 150,301 are potential MRESS SNPs. We calculated SNP density by dividing the total number of SNPs in a certain region by the total length of such region in unit of kilo bases.

### Predicting microRNA-mRNA interactions using TargetScan6.1

As most validated MRESSs are located in the 3′UTR of mature mRNAs, we only predicted MRESSs for the 29,962 3′UTR sequences. To evaluate the effects of SNPs on microRNA-mRNA interactions, we generated two groups of 3′UTR sequences: wild type sequences and mutant type sequences. Wild type sequences are just reference sequences retrieved from NCBI. Sequences with length less than 50 were discarded because of their low probabilities of being targeted by microRNA. To generate mutant type sequences, we first got the flanking region of 50 base long on both sides of each 3′UTR SNP, and then replaced base at the position of SNP with the mutant allele. For each group of the 3′UTR sequences, we predicted MRESS for both seed set 1–7 and seed set 2–8. MRESSs of seed set 1–7 were subdivided into 7mer (1–7) and 8mer sites according to their match length while MRESSs of seed set 2–8 were subdivided into 7mer (2–8), 8mer-1a and 8mer sites. A 8mer-1a site was a special case of the 7mer (2–8) site. It had exact match to position 2–8 of the mature microRNA and followed by an ‘A’. SNPs embedded in MRESSs that only present in wild type were classified as ‘loss’ while SNPs mapped to the mutant-only MRESSs were classified as ‘create’. We conducted global alignment for mRNAs shared by both human and mouse using the ‘pairwiseAlignment’ function provided by the ‘BSgenome’ package of R. Penalty for gap-opening is set to −10 and gap-extension is set to −4. Only MRESSs which entirely matched to the aligned regions were considered as conserved.

### Retrieval of GWAS datasets and p-value assignment of MRESS SNPs

We retrieved publicly available GWAS data from the NCBI dbGaP database by the following criteria: a) coming from a case–control study; b) with more than 1000 samples for both case group and control group in replication stage; c) samples are mainly European ancestry; d) with analysis that contains p-value for SNPs; e) studying disease or cancer that is common to human.

We utilized the ‘proxy search’ tool provided by the SNAP database to obtain SNPs having high LD with the predicted MRESS SNPs. We chose dataset 1000 genome pilot1 and set D’ threshold equal to 1. Other parameters were set with the default options. We matched MRESS SNPs and their proxies to each GWAS dataset, and inherited their p-values in GWAS. If the p-values were different between a MRESS SNP and its proxies, the minimum one was retained.

### Retrieval of disease-related genes

For each GWAS dataset we generated a list of disease-related genes integrating data from the database CTD, Gene Card and KEGG. For CTD, we searched the studied disease of each GWAS dataset in the ‘Diseases’ entry, and then clicked the ‘Genes’ tag and recorded all the genes with direct association evidence (labeled as ‘M’ or ‘T’). For Gene Card, we collected disease-associated genes in the ‘Genes Associated with Diseases’ table. For KEGG, we searched the disease names in the ‘KEGG DISEASE’ entry and marked the ‘gene’ checkbox at the right of the input box to obtain genes that participate in the disease pathway.

### Retrieval of microRNA-mRNA co-expression evidence

MirCoX database deposited microRNA-mRNA co-expression data generated by analyzing results of public available RNA-seq experiments. Each microRNA-mRNA co-expression pair had a calculated correlation coefficient. Negative correlation coefficient may imply real interaction. We downloaded the whole database in MySQL format, and then abstracted correlation data for all the predicted microRNA-mRNA interactions, including the interactions disrupted by candidate causal MRESS SNPs.

### Calculation of binding free energy

We calculated binding free energy by MELTING 5.1, which is designed to calculate the enthalpy, entropy and melting-temperature of the helix-coil transitions for a nucleic acid duplex. Binding free energy can be calculated according to the formula *Δ*G = *Δ*H–310.15 × *Δ*S. MELTING employs a nearest-neighbor algorithm, which can receive an accurate result when the duplex is relatively short. But it lacks the ability to calculate energy for duplex with terminal-mismatch. To overcome this disadvantage, we added three repetitively complementary base-pairs to both ends of each microRNA-mRNA duplex. As we only calculated the energy alternation, additional base-pairs will not affect the final result. Parameters we used for calculating are –E Na = 1, −P 1e-4, and -H rnarna.

## Electronic supplementary material

Additional file 1: **List of the 286 candidate causal MRESS SNPs.** List of the 286 candidate causal MRESS SNPs selected out using our method. Other information such as p-values in GWAS dataset is also provided. (XLS 94 KB)

## Abbreviations

SNPs: Single nucleotide polymorphisms
MRESSs: MicroRNA recognition element seed sites
GWAS: Genome-wide association study
OR: Odds ratio
GO: Gene ontology
SLE: Systemic lupus erythematosus
FMF: Familial mediterranean fever
FC: Fold change
ΔΔG: Alternation of binding free energy.
